# A novel *MT-CO2* variant causing cerebellar ataxia and neuropathy: The role of muscle biopsy in diagnosis and defining pathogenicity

**DOI:** 10.1016/j.nmd.2021.05.014

**Published:** 2021-11

**Authors:** Karen Baty, Maria E. Farrugia, Sila Hopton, Gavin Falkous, Andrew M. Schaefer, William Stewart, Hugh J. Willison, Mary M. Reilly, Emma L. Blakely, Robert W. Taylor, Yi Shiau Ng

**Affiliations:** a Wellcome Centre for Mitochondrial Research, Translational and Clinical Research Institute, Faculty of Medical Sciences, Newcastle University, Newcastle upon Tyne NE2 4HH, UK; b NHS Highly Specialised Services for Rare Mitochondrial Disorders, Newcastle upon Tyne Hospitals NHS Foundation Trust, Newcastle upon Tyne NE1 4LP, UK; c Department of Neurology, Institute of Neurological Sciences, Queen Elizabeth University Hospital, Glasgow G51 4TF, UK; d Directorate of Neurosciences, Royal Victoria Infirmary, Newcastle upon Tyne Hospitals NHS Foundation Trust, Newcastle upon Tyne NE1 4LP, UK; e Department of Neuropathology, Queen Elizabeth University Hospital, Glasgow G51 4TF and Institute of Neuroscience and Psychology, University of Glasgow, G12 8QQ, UK; f Department of Neurology and Institute of Infection, Immunity and Inflammation, University of Glasgow, Glasgow G51 4TF, UK; g MRC Centre for Neuromuscular Diseases, Department of Neuromuscular Diseases, UCL Queen Square Institute of Neurology, London, WC1N 3BG, UK

**Keywords:** Mitochondrial DNA, Muscle biopsy, Segregation study

## Abstract

Pathogenic variants in mitochondrial DNA (mtDNA) are associated with significant clinical heterogeneity with neuromuscular involvement commonly reported. Non-syndromic presentations of mtDNA disease continue to pose a diagnostic challenge and with genomic testing still necessitating a muscle biopsy in many cases. Here we describe an adult patient who presented with progressive ataxia, neuropathy and exercise intolerance in whom the application of numerous Mendelian gene panels had failed to make a genetic diagnosis. Muscle biopsy revealed characteristic mitochondrial pathology (cytochrome c oxidase deficient, ragged-red fibers) prompting a thorough investigation of the mitochondrial genome. Two heteroplasmic *MT-CO2* gene variants (NC_012920.1: m.7887G>A and m.8250G>A) were identified, necessitating single fiber segregation and familial studies – including the biopsy of the patient's clinically-unaffected mother - to demonstrate pathogenicity of the novel m.7887G>A p.(Gly101Asp) variant and establishing this as the cause of the mitochondrial biochemical defects and clinical presentation. In the era of high throughput whole exome and genome sequencing, muscle biopsy remains a key investigation in the diagnosis of patients with non-syndromic presentations of adult-onset mitochondrial disease and fully defining the pathogenicity of novel mtDNA variants.

## Highlights

• Non-syndromic presentations of mtDNA-related adult disease are diagnostically challenging.
• Access to diagnostic muscle biopsies from the patient and his clinically-unaffected mother were essential in defining which of two heteroplasmic *MT-CO2* variants identified through mitochondrial DNA sequencing were causal.
• Muscle biopsy remains a vital diagnostic investigation in the era of next generation sequencing.

## 1 Introduction

The phenotypic spectrum and genetic aetiology of mitochondrial disease are heterogeneous and the diagnostic challenges are well-recognized [1]. The increased application of whole exome sequencing (WES) and whole genome sequencing (WGS) as a first step in the diagnostic pathway has successfully identified many novel Mendelian mitochondrial genes or new pathogenic variants in known disease genes over the last decade [2,3]. In the adult population, around two thirds of patients with mitochondrial disease have pathogenic mitochondrial DNA (mtDNA) variants of which some of these variants cannot be reliably detected in blood [4]. Typical clinical phenotypes, such as chronic progressive external ophthalmoplegia (CPEO) [5] or multisystem involvement frequently prompt clinicians to consider mitochondrial disease and perform muscle biopsy to guide further genetic investigation. However, adult-onset, non-syndromic presentation of mtDNA disease remain difficult to diagnose because a high index of clinical suspicion is required to distinguish it [6,7] from other genetic mimics including autosomal recessive cerebellar ataxia and hereditary neuropathy [8]. A study from the ataxia specialist centers identified approximately a quarter of the patients with ‘idiopathic’ progressive cerebellar ataxia had canonical features of mitochondrial disease in their muscle biopsies, highlighting the utility of muscle biopsy should not be confined to the investigation of myopathy [9].

Here we report a patient who presented with adult-onset progressive ataxia, neuropathy and exercise intolerance without relevant family history. Extensive genetic testing had failed to provide a genetic diagnosis prior to muscle biopsy revealing marked mitochondrial histopathological changes. We detail the work-up of genetic investigations that identified two mtDNA variants in the same gene and how access to familial samples including the biopsy of his clinically-unaffected mother helped define the causal mtDNA genetic variant.

## 2 Methods

### 2.1 Case report

A 45-year-old man presented to the neurology clinic with slowly progressive sensory symptoms in the lower limbs and impaired balance since his early twenties. He also reported muscle pain after physical exertion but never noticed discoloured urine. He was unsteady when he walked and this became worse in the dark. He was never good at sport at school; he could run but avoided sports altogether since early teenage. Upper limb incoordination, slurred speech, worsening exercise intolerance and fatigue were reported in his thirties. As his unsteadiness progressed, he started to fall up to four times a week and began to use a walking stick when he was 40 years old. He suffered from chronic constipation since his mid-30 s; he also complained of erectile dysfunction but did not have any urinary symptoms. He was aware of mild hearing deficits since his late 20 s.

Other past medical history included anxiety and depression. His-birth history was unremarkable. He achieved all neurodevelopmental milestones other than he walked at a slightly older age (14 months) compared to his younger sister. There was no family history of neuromuscular disease, ataxia, diabetes or deafness.

The main clinical examination findings between aged 37 and 40 years old were dysarthric speech, upper and lower limb ataxia, broad-based gait and positive Romberg's sign. In addition, all the deep tendon reflexes were absent, there was evidence of reduced pin prick sensation following the stocking distribution and asymmetrical loss of vibration sense (left knee and right rib coastal margin, respectively). There was no eyelid ptosis, his eye movement was normal, and muscle tone and power were within the normal limit.

Routine laboratory investigations including normal vitamin B12 and folate, autoimmune screen, creatinine kinase, Vitamin A and E levels, were unremarkable. Serum lactate was marginally raised at 2.7 mmol/L (normal range up to 2.2 mmol/L). Urinary thymidine and uridine levels were within normal limits. Nerve conduction studies and electromyography, performed at the age of 35 years, identified axonal sensory neuropathy and mild neurogenic changes in muscles. A repeat study five years later showed little progression, with retained but reduced amplitudes in sensory nerve action potentials (SNAP) in all limbs (sural amplitude 2μV for both sides; and superficial peroneal amplitude 2μV on the right, absent on the left), and normal motor nerve function.

**Fig. 1:**
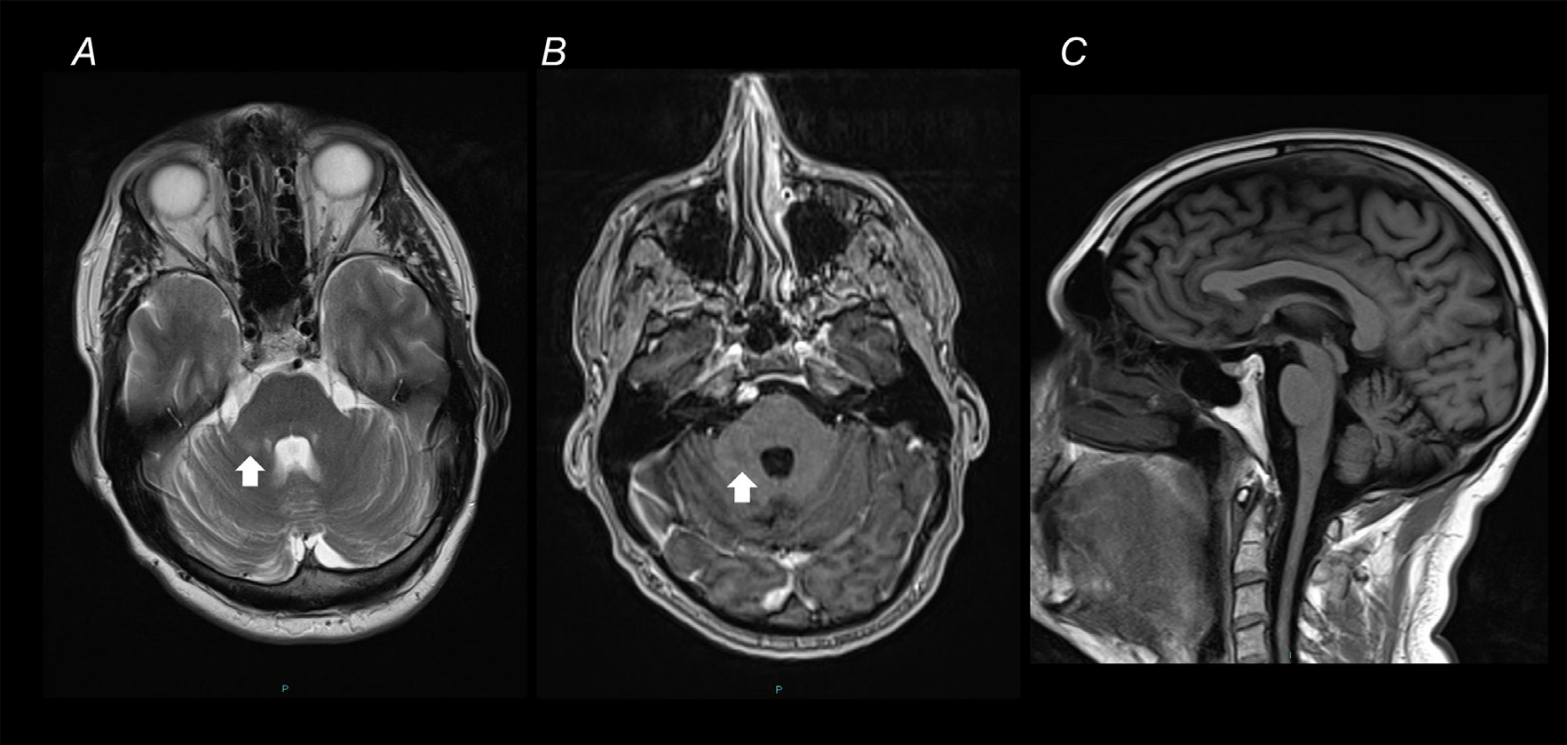
**Cranial MRI scans.** (**A**) Axial view T2-weighted sequence showing a hyperintensity in right middle cerebellar peduncle, without evidence of contrast enhancement (arrow) as shown in (**B**), the T1-weighted sequence. (**C**) Sagittal view T1 sequence showing mild cerebellar atrophy.

Given the complaint of muscle fatigue and exercise intolerance, blood spot and plasma acylcarnitine profiles were performed and showed increased medium to long chain acylcarnitine species. He reported some improvement of exercise intolerance with the supplementation of riboflavin and co-enzyme Q10. His-resting 12-lead electrocardiogram and transthoracic echocardiogram were normal. MRI head showed mild cerebellar atrophy and a T2 hyperintensity in the right cerebellar peduncle, without any contrast enhancement (Fig. 1). A detailed ophthalmological assessment identified pigmentary retinal changes and slight optic disc pallor.

The overall clinical picture was one of cerebellar ataxia with sensory neuropathy and pigmentary retinopathy. He was tested for trinucleotide expansions in common spinocerebellar ataxia (SCA) and frataxin genes, but no abnormalities were found; a screen of the *POLG* gene was normal. Several gene panels including Charcot Marie Tooth neuropathy type 2 (24 genes), autosomal dominant and recessive SCA (21 genes), hereditary sensory neuropathy (11 genes) and hereditary spastic paraplegia (12 genes) were also uninformative (Gene targets identified in Supplemental appendix).

In the absence of a genetic diagnosis, he underwent skeletal muscle biopsy of vastus lateralis at the age of 44 years. His-family members were reviewed and examined in the clinic to help with the segregation studies of identified genetic variants. The patient's mother was 72 years old at the time of review, her past medical history including breast cancer treated with mastectomy and chronic low back pain. Her neurological examination including fundoscopy was within normal limits. He has a younger sister (examined at the age of 42 years) who is fit and well and with a normal neurological assessment.

### 2.2 Histopathological analysis of the muscle biopsy

Standard histological (haematoxylin and eosin (H&E); Gomori trichrome; Sudan black) and histochemical (cytochrome c oxidase (COX), succinate dehydrogenase (SDH) and sequential COX/SDH activities) investigations were performed on frozen, transversely orientated skeletal muscle (10 µm) sections as described [10]. Additionally, quantitative, quadruple immunohistochemical (IHC) analyses were used to assess mitochondrial OXPHOS function, interrogating complex I (NDUFB8), complex IV (COX1) and porin (VDAC1; a mitochondrial mass marker) protein expression [11].

### 2.3 Mitochondrial genetic studies

Total DNA was extracted from skeletal muscle by standard procedures. Screening for the common m.3243A>G variant was undertaken using a quantitative pyrosequencing assay, while large scale mtDNA rearrangements were assessed using several long-range polymerase chain reaction (PCR) assays. The entire mitochondrial genome (GenBank Accession number: NC_012920.1) was amplified, analyzed and sequenced at a minimum read depth of 200x as previously reported [10].

### 2.4 Single fibre segregation analysis and mtDNA heteroplasmy assessment

Two mtDNA variants, m.7887G>A (novel) and m.8250G>A (extremely rare), were identified in the *MT-CO2* gene following the sequencing of the mitochondrial genome in the patient's muscle sample. These variants were investigated further in a broad range of tissues (skeletal muscle, urinary sediments, blood and buccal epithelia) and individual (COX-deficient and COX-positive) skeletal muscle fibers isolated by laser-capture microdissection using a quantitative pyrosequencing assay designed to accurately determine the level of heteroplasmy at these sites. The two novel variants were also investigated in non-invasive tissues (urinary sediments, blood and buccal epithelia) from the patient's mother and sister using this method. The assay was performed on a PyroMark Q24 platform (Qiagen) and used variant-specific primers (available from the authors on request). The assay can reliably detect to a level of >3% heteroplasmy. Data analysis was performed using PyroMark Q24 software (v2.0.7).

## 3 Results

### 3.1 Histochemistry and immunohistochemical analyses of patient muscle

**Fig. 2:**
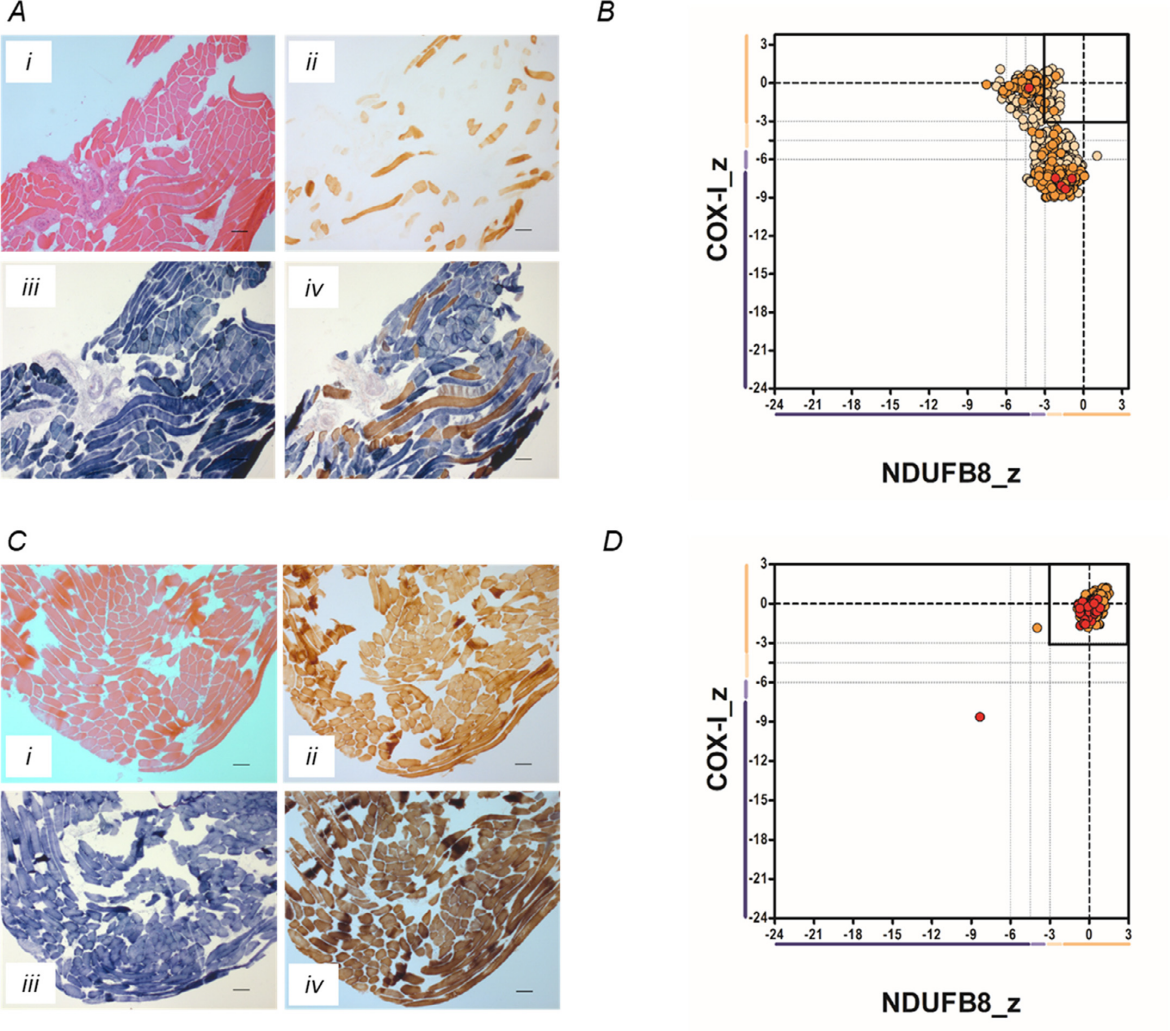
**Histopathological and immunohistochemical studies in muscle.** (**A**) Hematoxylin and eosin (H&E) staining (i), cytochrome *c* oxidase (COX) histochemistry (ii), succinate dehydrogenase (SDH) histochemistry (iii) and sequential COX-SDH histochemistry (iv) demonstrate a striking mosaic pattern of COX deficiency in patient muscle with several fibres showing abnormal, subsarcolemmal accumulation of mitochondria. Scale bar = 100 µm. (**B**) Quadruple immunofluorescence analysis of NDUFB8 (complex I) and COXI (Complex IV) protein expression in patient muscle. Each dot represents the measurement from an individual muscle fibre, colour co-ordinated according to its mitochondrial mass (low = blue, normal = beige, high = orange, very high = red). grey dashed lines represent SD limits for classification of the fibres. Lines next to x- and y- axis represent the levels of NDUFB8 and COXI: beige = normal (>−3), light beige = intermediate positive (−3 to −4.5), light purple = intermediate negative (−4.5 to −6), purple = deficient (<−6). Bold dashed lines represent the mean expression level of normal fibres. These data confirm the loss of COX1 expression in the majority of fibres, consistent with the molecular genetic defect identified. (**C** and **D**) Histopathological and immunohistochemical analyses of a muscle biopsy from the patient's clinically-unaffected mother essentially shows a normal pattern of activity and protein expression; the focal changes observed in (**D**) are likely to be due to age-related, somatic mtDNA mutation. (For interpretation of the references to color in this figure legend, the reader is referred to the web version of this article.).

Histological and histochemical assessment of the skeletal muscle sections showed evidence of mitochondrial dysfunction following sequential COX/SDH histochemistry, demonstrating ∼80% COX-deficient fibers (Fig. 2**A**) as well as ragged-red fibers in the Gomori trichrome and excess lipid accumulation. IHC analyses confirmed extensive mitochondrial dysfunction, >60% of all fibres analysed (*n* = 1030), showing complete loss of complex IV protein expression (Fig. 2**B**). A muscle biopsy sample from the patient's clinically-unaffected mother revealed normal histopathological (Fig. 2**C**) and immunohistochemical (Fig. 2**D**) profiles.

### 3.2 Mitochondrial genetic studies

**Fig. 3:**
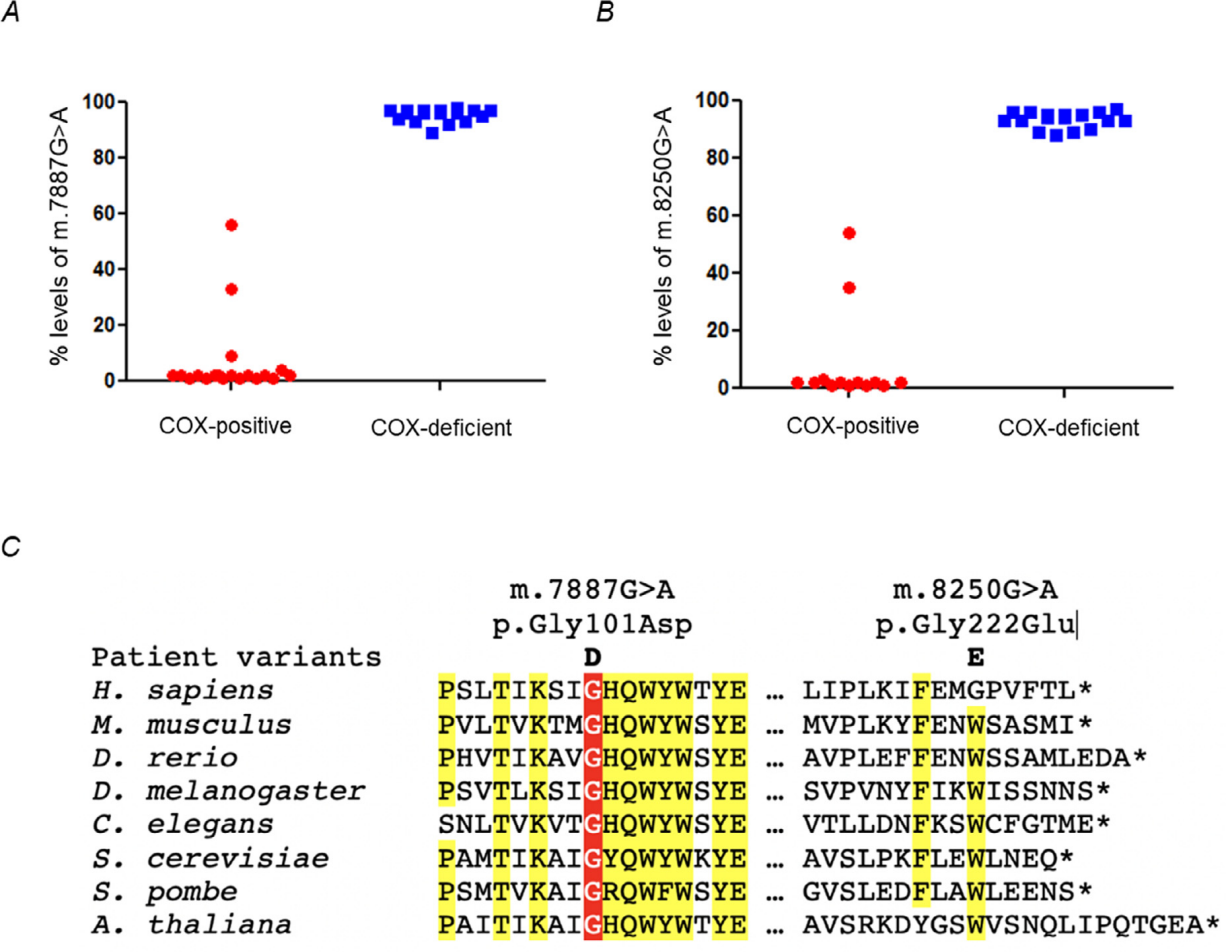
**Molecular genetic analysis of novel *MT-CO2* variants.** (**A**) and (**B**) show near identical segregation patterns of the respective m.7887G>A and m.8250G>A variants in individual COX-deficient and COX-positive muscle fibres from the patient, confirming segregation of high mutant loads with a biochemical defect, suggesting both variants are present on identical mtDNA molecules. (**C**) Multiple sequence alignment of the COX2 protein sequence highlighting the strong evolutionary conservation of p.(Gly101) whilst p.(Gly222) shows weak evolutionary conservation and is located at the C-terminal end of the protein sequence.

No evidence of the common pathogenic m.3243A>G variant or large-scale mtDNA rearrangements was detected prior to sequencing of the entire mitochondrial genome. Analysis of the mitochondrial genome in patient muscle revealed two novel variants: m.7887G>A p.(Gly101Asp) and m.8250G>A p.(Gly222Glu), and both at high levels of heteroplasmy (78% mutant load). The m.7887G>A variant is not listed on publicly available databases including GenBank (51,836 human mtDNA sequences interrogated on 15th Jan 2021) or our own in-house database comprising >2500 mtDNA sequences, and the m.8250G>A variant is extremely rare. The m.7887G>A variant predicts the substitution of a highly-conserved, hydrophobic glycine residue with an aspartic acid residue, whilst the m.8250G>A variant is predicted to substitute a weakly-conserved glycine residue with a glutamic acid residue (Fig. 3).

Both these variants showed lower levels of heteroplasmy in a range of tissues (urinary sediment, blood and buccal epithelia). The m.7887G>A variant was present at levels of 21%, 6% and 3% respectively; the m.8250G>A variant was detected at levels of 17% and 5% in urinary sediment and blood, although was undetectable (within the limits of sensitivity of the assay) in buccal epithelia derived DNA.

Single muscle fibre segregation studies in individual COX-positive and COX-deficient fibres revealed that both variants segregated to COX-deficient fibres suggesting that both variants co-exist on the same mtDNA molecule. For the m.7887G>A variant, analysis of 19 COX-positive fibres showed a mean mutation load of 7 ± 3%, whilst 17 COX-deficient fibres showed a mean mutation load of 95 ± 1% (unpaired *t-*test *p* < 0.0001) and for the m.8250G>A variant, analysis of 13 COX-positive fibres showed a mean mutation load of 8 ± 5%, whilst 17 COX-deficient fibres showed a mean mutation load of 93 ± 1% (unpaired *t*-test *p* < 0.0001) (Fig. 3).

Quantitative pyrosequencing of non-invasive tissue samples taken from the patient's mother revealed the m.8250G>A variant to be present at low levels in urinary sediment and buccal epithelia-derived DNA (16% and 5% respectively) and at intermediate levels in blood (36%). However, the m.7887G>A variant was undetectable in urinary sediment, blood and buccal epithelia derived samples. She underwent a muscle biopsy at aged 72 years old; the m.7887G>A was not detectable but the m.8250G>A was present at high levels (87%); in the context of her normal muscle histopathology (Fig. 2), this strongly implicates the m.7887G>A variant as causal. Interestingly, both variants were undetectable in blood, urinary sediment and buccal-epithelia-derived DNA of the patient's sister.

## 4 Discussion

We describe a 45-year-old man presenting with progressive cerebellar syndrome with sensory neuropathy with onset in his 20 s. Extensive genetic screening failed to identify a genetic cause, prompting diagnostic muscle biopsy, which indicated an underlying mitochondrial aetiology. Mitochondrial genome sequencing in muscle revealed two mtDNA variants - m.7887G>A (novel) and m.8250G>A (extremely rare) – predicting missense variants in the *MT-CO2* gene, compatible with the marked mitochondrial histopathological abnormalities. Although both variants attain some measures proposed for a pathogenic mtDNA variant, our wider investigations strongly suggest that only the m.7887G>A; p.(Gly101Asp) variant completely fulfills criteria necessary to prove causality of the patient's clinical phenotype.

First, the m.7887G>A variant is absent on large, publicly accessible mitochondrial genetic databases and m.8250G>A is extremely rare. Second, both mtDNA variants are heteroplasmic and demonstrate the highest mutant loads in clinically-affected, postmitotic skeletal muscle, with lower levels in urinary epithelia, blood and buccal cells. Third, histochemical studies of the patient's muscle biopsy showed a dramatic loss of COX activity, with quantitative, quadruple OXPHOS immunohistochemistry showing a consistent level of loss of COXI protein expression (Fig. 2); both findings would be expected for and predicted by a pathogenic *MT-CO2* variant. Fourth, single fibre segregation studies established that both variants segregate with the COX histochemical defect in skeletal muscle with higher levels of mutant load in COX-deficient fibres. However, there are two lines of additional evidence to support the pathogenicity of m.7887G>A variant. The m.7887G>A variant was found to have arisen *de novo* and absent in all tissues of other maternal family members investigated. There was no evidence of COX deficiency in the muscle tissue of patient's mother who harbors a higher heteroplasmy of m.8250G>A variant and she is clinically unaffected. Moreover, the m.7887G>A variant predicts the substitution of a highly-conserved, hydrophobic glycine residue with an aspartic acid residue in a region of the COX2 protein that shows strong evolutionary conservation, whilst the m.8250G>A variant predicts the change of a poorly-conserved amino acid at the very C-terminal end of the protein (Fig. 2**C**).

Our case study also illustrates several diagnostic caveats to underpin the diagnosis of mtDNA disease in clinical practice. The genetic aetiology of non-syndromic ataxia with axonal neuropathy is highly heterogeneous [12,13] with a Mendelian pattern of recessive inheritance often assumed in the absence of any relevant family history. Recent studies have suggested that *de novo* mtDNA variants are not uncommon [14] with over half of all reported pathogenic *MT-CO2* variants having arisen *de novo* [7]. Pathogenic *MT-CO2* variants have been linked to several clinical phenotypes with different disease onset, such as myopathy with [15] or without [16] recurrent myoglobinuria, multisystem disease characterized by neurodevelopmental delay, gait disorder, cardiac involvement, retinitis pigmentosa and lactic acidosis [17,18], MELAS syndrome [19] and progressive cerebellar ataxia [7]. WES and WGS are increasingly integrated with mainstream genetic investigations in a “genetics first” approach and their abilities of detecting pathogenic mtDNA variants have been widely demonstrated [20,21]. However, the complexity of assigning pathogenicity of any mtDNA alterations is further complicated by the presence of mtDNA heteroplasmy as well as skewed tissue segregation of mtDNA variants. Low mutant heteroplasmies detected in EDTA-blood DNA samples can be inadvertently overlooked or regarded as incidental in some diagnostic pipelines. In agreement with earlier reports [6,^7^,^22^,23], muscle biopsy remains crucial in the diagnostic algorithm of adult-onset mitochondrial disease, particularly when evaluating the pathogenicity of novel mtDNA variants [24].

We identified the accumulation of medium-chain acylcarnitine species in the patient's plasma, similar to the findings reported in other pathogenic *MT-CO2* variants [7,^15^,16], which mimic the findings in patients with primary fatty acid oxidation disorder due to multiple acyl-CoA dehydrogenase deficiency (MADD). Abnormal acylcarnitine profiles in the fibroblasts of patients with mitochondrial respiratory chain deficiencies, resembling patients with various primary fatty acid oxidation defects, were previously reported [25]. More recently, a metabolomics study of nine patients with LRPPRC-related mitochondrial disease (deficiency in this nuclear-encoded mitochondrial protein also causes isolated complex IV deficiency) identified elevated C2, C6, C12, C14, C14:1, C16, C18:1, and C18:2 acylcarnitines, suggesting the perturbation of fatty acid oxidation pathway is likely to be secondary to mitochondrial respiratory chain dysfunction [26].

Co-segregation of two (possible) pathogenic mtDNA variants has occasionally been reported in the literature. In some cases, the co-presence of two pathogenic mtDNA variants was thought to contribute the clinical manifestations, such as identification of m.11778G>A and a single, large-scale mtDNA deletion – the so-called “common mtDNA deletion” - in a young man who with childhood-onset CPEO who later developed symptomatic, bilateral optic neuropathy [27]; co-existence of single, large-scale deletion and m.3243A>G has also been reported in a woman with Kearns-Sayre syndrome and multiple endocrine disorder [28]. The exact roles of co-segregated mtDNA variants cannot be robustly determined in some patients, leading to speculation of a synergistic, pathogenic effect or that one of the mtDNA variants may act to modulate the penetrance of the second mtDNA variant [29], [30], [31]–32]. On the other hand, some of these variants can be classified as neutral polymorphisms retrospectively when more stringent diagnostic criteria have been applied [33], [34]–35]. Our case study is unique as we have established that only the de novo m.7887G>A variant is pathogenic, whilst the maternally-inherited m.8250G>A variant is a polymorphism through the analysis of the patient's and his mother's muscle biopsies. Based on these findings, we have been able to provide genetic counseling to other maternal family members.

In conclusion, we describe a novel pathogenic m.7887G>A p.(Gly101Asp) *MT-CO2* gene variant in a patient who manifests with an ataxia neuropathy spectrum mimicking autosomal recessive cerebellar ataxia and highlight the continuing requirement for diagnostic muscle biopsy in cases of adult-onset mtDNA disease, particularly those necessitating the characterization of novel sequence variants [22].

## Declaration of Competing Interest

Nothing to declare.

## Appendix Supplementary materials

Image, application 1

## References

[1] La Morgia C., Maresca A., Caporali L., Valentino M.L., Carelli V (2020). Mitochondrial diseases in adults. J Intern Med.

[2] Thompson K., Collier J.J., Glasgow R.I.C., Robertson F.M., Pyle A., Blakely E.L. (2020). Recent advances in understanding the molecular genetic basis of mitochondrial disease. J Inherit Metab Dis.

[3] Stenton S.L., Prokisch H. (2020). Genetics of mitochondrial diseases: identifying mutations to help diagnosis. EBioMedicine.

[4] Gorman G.S., Schaefer A.M., Ng Y., Gomez N., Blakely E.L., Alston C.L. (2015). Prevalence of nuclear and mitochondrial DNA mutations related to adult mitochondrial disease. Ann Neurol.

[5] Rodríguez-López C., García-Cárdaba L.M., Blázquez A., Serrano-Lorenzo P., Gutiérrez-Gutiérrez G., San Millán-Tejado B. (2020). Clinical, pathological and genetic spectrum in 89 cases of mitochondrial progressive external ophthalmoplegia. J Med Genet.

[6] Ng Y.S., Thompson K., Loher D., Hopton S., Falkous G., Hardy S.A. (2020). Novel MT-ND gene variants causing adult-onset mitochondrial disease and isolated complex I deficiency. Front Genet.

[7] Zierz C.M., Baty K., Blakely E.L., Hopton S., Falkous G., Schaefer A.M., Sarrigiannis PG, Ng YS, Taylor RW (2019 Jun 4). A Novel Pathogenic Variant in MT-CO_2_ Causes an Isolated Mitochondrial Complex IV Deficiency and Late-Onset Cerebellar Ataxia. J Clin Med.

[8] Renaud M., Tranchant C., Martin J.V.T., Mochel F., Synofzik M., van de Warrenburg B. (2017). A recessive ataxia diagnosis algorithm for the next generation sequencing era. Ann Neurol.

[9] Bargiela D., Shanmugarajah P., Lo C., Blakely E.L., Taylor R.W., Horvath R. (2015). Mitochondrial pathology in progressive cerebellar ataxia. Cerebellum Ataxias.

[10] Old S.L., Johnson M.A. (1989). Methods of microphotometric assay of succinate dehydrogenase and cytochrome c oxidase activities for use on human skeletal muscle. Histochem J.

[11] Rocha M.C., Grady J.P., Grunewald A., Vincent A., Dobson P.F., Taylor R.W. (2015). A novel immunofluorescent assay to investigate oxidative phosphorylation deficiency in mitochondrial myopathy: understanding mechanisms and improving diagnosis. Sci Rep.

[12] Beaudin M., Matilla-Dueñas A., Soong B.W., Pedroso J.L., Barsottini O.G., Mitoma H. (2019). The classification of autosomal recessive cerebellar ataxias: a consensus statement from the society for research on the cerebellum and ataxias task force. Cerebellum.

[13] Rossi M., Anheim M., Durr A., Klein C., Koenig M., Synofzik M. (2018). The genetic nomenclature of recessive cerebellar ataxias. Mov Disord Off J Mov Disord Soc.

[14] Sallevelt S.C., de Die-Smulders C.E., Hendrickx A.T., Hellebrekers D.M., de Coo I.F., Alston C.L., Knowles C, Taylor RW, McFarland R, Smeets HJ (2017). De novo mtDNA point mutations are common and have a low recurrence risk. J Med Genet.

[15] Vissing C.R., Duno M., Olesen J.H., Rafiq J., Risom L., Christensen E. (2013). Recurrent myoglobinuria and deranged acylcarnitines due to a mutation in the mtDNA MT-CO2 gene. Neurology.

[16] Roos S., Sofou K., Hedberg-Oldfors C., Kollberg G., Lindgren U., Thomsen C. (2019). Mitochondrial complex IV deficiency caused by a novel frameshift variant in MT-CO2 associated with myopathy and perturbed acylcarnitine profile. Eur J Hum Genet EJHG.

[17] Horvath R., Schoser B.G., Muller-Hocker J., Volpel M., Jaksch M., Lochmuller H. (2005). Mutations in mtDNA-encoded cytochrome c oxidase subunit genes causing isolated myopathy or severe encephalomyopathy. Neuromus Disord NMD.

[18] Clark K.M., Taylor R.W., Johnson M.A., Chinnery P.F., Chrzanowska-Lightowlers Z.M., Andrews R.M. (1999). An mtDNA mutation in the initiation codon of the cytochrome C oxidase subunit II gene results in lower levels of the protein and a mitochondrial encephalomyopathy. Am J Hum Genet.

[19] Rossmanith W., Freilinger M., Roka J., Raffelsberger T., Moser-Thier K., Prayer D. (2008). Isolated cytochrome c oxidase deficiency as a cause of MELAS. J Med Genet.

[20] Wei W., Tuna S., Keogh M.J., Smith K.R., Aitman T.J., Beales P.L. (2019). Germline selection shapes human mitochondrial DNA diversity. Science.

[21] Wagner M., Berutti R., Lorenz-Depiereux B., Graf E., Eckstein G., Mayr J.A. (2019). Mitochondrial DNA mutation analysis from exome sequencing-a more holistic approach in diagnostics of suspected mitochondrial disease. J Inherit Metab Dis.

[22] Hardy S.A., Blakely E.L., Purvis A.I., Rocha M.C., Ahmed S., Falkous G. (2016). Pathogenic mtDNA mutations causing mitochondrial myopathy: the need for muscle biopsy. Neurol Genet.

[23] Lim A.Z., McMacken G., Rastelli F., Oláhová M., Baty K., Hopton S. (2020). A novel, pathogenic dinucleotide deletion in the mitochondrial MT-TY gene causing myasthenia-like features. Neuromusc Disord NMD.

[24] de Silva R.N., Vallortigara J., Greenfield J., Hunt B., Giunti P., Hadjivassiliou M. (2019). Diagnosis and management of progressive ataxia in adults. Pract Neurol.

[25] Sim K.G., Carpenter K., Hammond J., Christodoulou J., Wilcken B. (2002). Acylcarnitine profiles in fibroblasts from patients with respiratory chain defects can resemble those from patients with mitochondrial fatty acid beta-oxidation disorders. Metab Clin Exp.

[26] Thompson Legault J., Strittmatter L., Tardif J., Sharma R., Tremblay-Vaillancourt V., Aubut C. (2015). A metabolic signature of mitochondrial dysfunction revealed through a monogenic form of Leigh syndrome. Cell Rep.

[27] Melberg A., Moslemi A.R., Palm O., Raininko R., Stålberg E., Oldfors A. (2009). A patient with two mitochondrial DNA mutations causing PEO and LHON. Eur J Med Genet.

[28] Ohno K., Yamamoto M., Engel A.G., Harper C.M., Roberts L.R., Tan G.H. (1996). MELAS- and Kearns-Sayre-type co-mutation [corrected] with myopathy and autoimmune polyendocrinopathy. Ann Neurol.

[29] Meulemans A., De Paepe B., De Bleecker J., Smet J., Lissens W., Van Coster R. (2007). Two novel mitochondrial DNA mutations in muscle tissue of a patient with limb-girdle myopathy. Arch Neurol.

[30] Bortot B., Barbi E., Biffi S., Angelini C., Faleschini E., Severini G.M. (2009). Two novel cosegregating mutations in tRNAMet and COX III, in a patient with exercise intolerance and autoimmune polyendocrinopathy. Mitochondrion.

[31] Bacalhau M., Simões M., Rocha M.C., Hardy S.A., Vincent A.E., Durães J. (2018). Disclosing the functional changes of two genetic alterations in a patient with Chronic Progressive External Ophthalmoplegia: report of the novel mtDNA m.7486G>A variant. Neuromusc Disord.

[32] Swalwell H., Blakely E.L., Sutton R., Tonska K., Elstner M., He L. (2008). A homoplasmic mtDNA variant can influence the phenotype of the pathogenic m.7472Cins MTTS1 mutation: are two mutations better than one?. Eur J Hum Genet EJHG.

[33] Mimaki M., Ikota A., Sato A., Komaki H., Akanuma J., Nonaka I. (2003). A double mutation (G11778A and G12192A) in mitochondrial DNA associated with Leber's hereditary optic neuropathy and cardiomyopathy. J Hum Genet.

[34] Wong L.J.C., Chen T., Wang J., Tang S., Schmitt E.S., Landsverk M. (2020). Interpretation of mitochondrial tRNA variants. Genet Med.

[35] Bidooki S.K., Johnson M.A., Chrzanowska-Lightowlers Z., Bindoff L.A., Lightowlers R.N. (1997). Intracellular mitochondrial triplasmy in a patient with two heteroplasmic base changes. Am J Hum Genet.

