# Supplementary material for: A novel MT-CO2 variant causing cerebellar ataxia and neuropathy: The role of muscle biopsy in diagnosis and defining pathogenicity
Source: Neuromuscul Disord. 2021 Nov;31(11):1186–93. doi: 10.1016/j.nmd.2021.05.014 (PMC8708152; doi:10.1016/j.nmd.2021.05.014)
Supplement: Supplementary file 1 [file mmc1.docx]

**Supplemental Appendix**

| **Panel** | **Genes** |
| --- | --- |
| Hereditary spastic paraplegia (HSP) | *CYP7B1, FA2H, GJC2, KIAA0196, KIF5A, NIPA1, PLP1, PNPLA6, REEP1, RTN2, SPAST* and *SPG7.* |
| Autosomal dominant and recessive SCA gene panel | *ADCK3, ANO10, APTX, ATM, DNMT1, FGF14, FXN, GFAP, GJC2, ITPR1, KCNC3, KCND3, PDYN, PNPLA7, POLG, POLR3A, PRKCG, PRNP, SACS, SETX* and *TTBK2.* |
| Charcot-Marie-Tooth (CMT) neuropathy Type 2 | *AARS, BSCL2, DNM2, DYNC1H1, GARS, GDAP1, GJB1, HINT1, HSPB1, HSPB8, IGHMBP2, LMNA, LRSAM1, MARS, MFN2, MPZ, NEFL, PMP22, PRPS1, RAB7A, SH3TC2, TRPV4, VCP* and *YARS.* |
| Hereditary sensory neuropathy | *ATL1, CCT5, DNMT1, FAM134B, NGF, NTRK1, RAB7A, SCN9A, SPTLC1, SPTLC2* and *WNK1.* |
| - | Two normal FXN GAA alleles |
| SCA trinucleotide repeat disorder | *SCA1, SCA2, SCA3, SCA6* and *SCA7* |

Table. Summary of the nuclear genetic investigations.
